# PERSON-CENTERED CARE PLANNING FOR PERSONS WITH MCC: LESSONS FROM AN ENVIRONMENTAL SCAN

**DOI:** 10.1093/geroni/igae098.1860

**Published:** 2024-12-31

**Authors:** Annette Totten, Chandler Atchison, Cynthia Davis-O’Reilly, Katherine D Peak, Tamar Wyte-Lake, Deborah Cohen, Ana Quiñones

**Affiliations:** Oregon Health & Science University, Portland, Oregon, United States; Oregon Health & Science University, Portland, Oregon, United States; Oregon Health & Science University, Portland, Oregon, United States; Oregon Health & Science University, Portland, Oregon, United States; Oregon Health & Science University, Portland, Oregon, United States; Oregon Health & Science University, Portland, Oregon, United States; Oregon Health & Science University, Portland, Oregon, United States

## Abstract

To guide overall project objectives of describing the current state of the field of person-centered care planning (PCCP) for persons with MCC, the environmental scan functions as a multi-source overview of the field and integrates input from key informant interviews (KII) of experts from diverse perspectives. The objective is to clarify our understanding of which PCCP models have been implemented in the US and which show promise for improving outcomes for patients, their caregivers, and care teams. This task involves conducting one baseline, two ad hoc scans, and 15key informant interviews. We incorporate input from a technical expert panel to inform our literature search and augment synthesis with KIIs. KIIs provide greater context on the adoption and use of PCCP models in clinical settings, and features that may be important from a patient, clinic, or policy standpoint. KII analysis follows a five-step process: Describing, Organizing, Connecting, Corroborating/legitimating, Representing the account. The methods for the environmental scan are based on systematic review methods, but are modified to fit our rapid review timeline. Modifications include: limiting citation database searches and prioritizing MEDLINE first; prioritizing triage of abstracts and using one reviewer; focusing data abstraction on project partner priorities; foregoing risk of bias, strength of evidence, and quantitative synthesis in favor of targeted summaries. Analysis focuses on understanding successful components of and barriers to PCCP model implementation, and to identify what is required for successful implementation and sustainability.

